# Decoding the Potential Impact of Plasma hsa-miR-24-3p and hsa-miR-181 d-3p Expression, Plasma IFN-γ Levels, and *IFNG* rs2069727 T/C Genetic Variant on Multiple Sclerosis Risk and Glatiramer Acetate Treatment

**DOI:** 10.1007/s12035-025-05027-9

**Published:** 2025-06-02

**Authors:** Mekselina Kalmaz, Semra Mungan, Birsen Can Demirdöğen

**Affiliations:** 1 Department of Biomedical Engineering, TOBB University of Economics and Technology, Söğütözü, 06560 Ankara, Türkiye; 2 Department of Neurology, University of Health Sciences, Ankara Bilkent City Hospital, Ankara, Türkiye

**Keywords:** Multiple sclerosis, *IFNG* rs2069727 T/C, Interferon-γ, MicroRNA, Biomarker

## Abstract

Multiple sclerosis (MS) is an autoimmune neurodegenerative disorder, with relapsing–remitting MS (RRMS) being the most common subtype. Interferon-γ (IFN-γ) plays a dual role in MS pathogenesis. MicroRNAs (miRNAs) have emerged as potential diagnostic biomarkers. This study examined the effect of relative expression of hsa-miR-24-3p and hsa-miR-181d-3p, plasma IFN-γ levels, and the *IFNG* rs2069727 T/C variant on MS risk, evaluating their interrelationships and diagnostic potential. This case–control study comprised two overlapping groups—a genetic polymorphism group (330 RRMS, 330 healthy controls (HCs)) and a miRNA group (25 glatiramer acetate (GA)-treated RRMS patients, 25 treatment-naïve RRMS patients, and 25 HCs)- collected at the Ankara Bilkent City Hospital Neurology Polyclinic. The *IFNG* rs2069727 T/C variant did not display a statistically significant disparity between RRMS patients and HCs. Significantly elevated hsa-miR-24-3p and hsa-miR-181d-3p relative expression levels were observed in GA-treated and treatment-naïve RRMS patients compared to HCs. Conversely, age-adjusted plasma IFN-γ concentrations were markedly lower in GA-treated and treatment-naïve RRMS patients versus HCs. Individuals with low plasma IFN-γ levels (≤ 1.311 pg/mL) demonstrated significantly elevated hsa-miR-24-3p relative expression compared to the high IFN-γ group (> 1.311 pg/mL). Conversely, subjects with low hsa-miR-181d-3p levels (≤ 2.90) exhibited significantly higher plasma IFN-γ concentrations relative to those with high hsa-miR-181d-3p levels (> 2.90). In the GA-treated group, EDSS negatively correlated with age-adjusted plasma IFN-γ. This study identified age-adjusted plasma IFN-γ, hsa-miR-24-3p, and hsa-miR-181d-3p expression as potential blood-based biomarkers for RRMS diagnosis and analyzed them alongside disability scores. The miRNAs in this study can be further evaluated as prospective therapeutic targets.

## Introduction

Multiple sclerosis (MS) is an autoimmune disease that affects the central nervous system (CNS) through the formation of plaque-like lesions and variable degrees of neurodegeneration caused by inflammation and demyelination [1]. The etiology of MS remains unclear [1–3]; however, it can be considered a multifactorial disease and includes a genetic predisposition combined with environmental factors, such as infection with certain viruses, vitamin D deficiency, smoking, obesity, excessive salt intake, and epigenetic factors, including histone modifications, DNA methylation, and small noncoding RNAs (ncRNAs) [2–6]. Complex interactions among genetic [3], gene‒environment [2], and gene‒epigenome factors [3, 4], along with intergenerational epigenetic inheritance, account for over 55% of MS heritability [2]. Further research into these networks and new genetic and epigenetic mechanisms is essential [2–4, 6].

When considering clinical classifications of this disease, it should be noted that categorizing MS into distinct types is a simplification. MS is now understood to exist along a phenotypic continuum, reflecting the broad heterogeneity in its emergence and progression across individuals [7]. The clinical forms of MS can be classified into different groups, of which relapsing–remitting MS (RRMS) is the most common (85%) [1, 8]. Patients with RRMS experience attacks at different intervals, and complete or near-complete recovery is observed in the period between attacks [8]. No definitive treatment can completely eradicate MS. Glatiramer acetate (GA) is an immunomodulating agent used for treating RRMS [8, 9]. The mechanism of action of GA is based on supporting the shift from T helper 1 (Th1) to Th2 [9]. The pathogenesis of MS is rather complex [1, 8, 9]; however, several lines of evidence suggest that intense immune activation in MS pathophysiology occurs because of the relationships between pro-inflammatory and anti-inflammatory cytokines [10]. IFN-γ is a cytokine [11] whose importance in the immune system derives from its capacity for immunostimulation and immunomodulation [12]. However, its role in MS remains controversial, with evidence suggesting both detrimental [11, 12] and protective [13–15] effects.

Genetic variants and microRNAs (miRNAs) may influence the expression or function of IFN-γ, and thus, these factors should be more extensively investigated for their effects on susceptibility, disease course, and severity of MS [14]. According to a study conducted in a heterogeneous group of patients with RRMS and secondary progressive MS (SPMS), a form of MS that transition from relapsing–remitting to a steadily progressive course, the rs2069727 T/C variation in the *IFNG* gene is associated with MS risk, a relationship observed only in male MS patients. Specifically, men carrying the G allele for this single nucleotide polymorphism (SNP) had a greater expression level than those who did not [16]. The rs2069727 T/C SNP is located near miRNA binding sites and has been consistently identified as the genetic variation that most notably affects gene expression among *IFNG* genetic variations [16]. It is well established that allele frequencies vary among populations, and MS exhibits a polygenic inheritance pattern [8]. Therefore, in our study, the *IFNG* rs2069727 T/C SNP was investigated for the first time in our population regarding MS risk.

miRNAs are approximately 20–22 nucleotide long, single-stranded ncRNAs that affect gene expression post-transcriptionally, and their stability in body fluids is quite high; therefore, they have strong biomarker potential [17]. Serum and plasma are relatively easily accessible, convenient, semi-invasive biological samples that have been well-studied for treating various diseases, such as MS [18]. Among miRNAs that have target regions in the 3'UTR of the *IFNG* gene [19], miR-24-3p and miR-181 d-3p have been reported as negative regulators of *IFNG* expression through post-transcriptional control [20]. While miR-24-3p has been implicated in various biological processes such as cell proliferation, apoptosis, immune responses, and gene expression [21], miR-181 has been associated with cellular growth, development, and activation [22].

Early detection and treatment of MS can help delay its progression. Disease prognosis is generally assessed using the Expanded Disability Status Scale (EDSS) and MS Severity Scale (MSSS) [8]. However, these scores mainly measure physical disability and may not provide comprehensive indicators for disease prognosis [23]. Thus, molecular biomarkers measured in easily accessible biological fluids such as blood are needed to monitor the treatment process of patients and evaluate their response to treatment and the progression of the disease [17].

This study aimed to examine the plasma level of IFN-γ and plasma relative expression levels of hsa-miR-24-3p and hsa-miR-181 d-3p, and distribution of the *IFNG* rs2069727 T/C variation which are thought to influence IFN-γ levels, in terms of their contributions to MS risk and disability scores in treatment-naïve RRMS patients as well as in those receiving GA therapy. The relationships among these parameters were investigated to assess whether their combined association with the disease was more robust, and to assess their utility as biomarkers.

## Materials and Methods

### Patients and Healthy Controls

In this single-blind observational study, two overlapping groups were established: the genetic polymorphism study group and the miRNA and ELISA study group. For the genetic polymorphism section, 330 patients with RRMS and 330 healthy controls (HCs) were included in our study. From this group, 25 RRMS patients who had not yet initiated drug therapy (treatment-naïve), 25 RRMS patients receiving GA treatment, and 25 HCs were selected for the miRNA and ELISA study subgroup. The subgroup composition was influenced by the study’s focus on GA, a first-line treatment relevant to biomarker studies during early diagnosis [8] and its known impact on IFN-gamma expression [24]. Additionally, plasma samples required for miRNA analysis could not be obtained from all 660 participants. A total of 660 subjects—330 patients with RRMS and 330 HCs—were recruited from the Ankara Bilkent City Hospital, Neurology Outpatient Clinic, Ankara, Turkey. After obtaining approval from the hospital ethics committee (date: May 26, 2021, decision no: E1-21–1805) and written informed consent from the participants, the study was carried out according to the principles of the Declaration of Helsinki. The study participants were adults from an admixed Turkish population who were unrelated individuals. Subjects with other autoimmune diseases, cerebrovascular disease, acute infection, inflammatory diseases, diabetes mellitus, rheumatologic diseases, end-stage renal failure or replacement therapy, carcinoma, obesity, or pregnancy were excluded.

Diagnosis of MS was carried out by an experienced neurologist using the updated 2017 McDonald criteria [25] by clinical or magnetic resonance imaging (MRI) evidence that demonstrated two or more episodes at least 1 month apart (dissemination in time) and two or more lesions at separate regions in the CNS (dissemination in space). In some cases, oligoclonal bands in the cerebrospinal fluid were investigated to verify the diagnosis. Patients with RRMS were diagnosed based on their clinical course, which consisted of relapses with stable neurological impairment in between. Patients were clinically evaluated for EDSS [26], a scale of disability in MS, at disease onset and when included in this study (current EDSS). The MSSS, which considers the current EDSS score and the disease duration, was also evaluated [27].

HCs included in the genetic polymorphism study (n = 330) and the miRNA and ELISA study (n = 25) were composed of volunteers who presented to the Neurology Clinic at Ankara Bilkent City Hospital with complaints unrelated to MS, met the inclusion criteria, and had no diagnosis of MS or other neurodegenerative diseases. The HC group had no symptoms of MS or any other autoimmune disorder, cerebrovascular disease, cardiovascular disease, carcinoma, acute infection, inflammatory disease, rheumatologic disease, trauma, end-stage renal failure, or replacement therapy.

Age, sex, smoking status, and clinical information, including duration of disease, duration of treatment, presence of contrast-enhancing lesions, and fasting serum lipid parameters, were collected. Also, after diagnosis or initiation of treatment, patients received 25-hydroxyvitamin D_3_ supplements under the doctor's supervision. For the genetic polymorphism study, peripheral blood samples were collected into tubes containing Na-EDTA and stored at − 86℃ until analysis. For the measurement of plasma levels of hsa-miR-24-3p and hsa-miR-181 d-3p and plasma IFN-γ parameters, additional peripheral blood samples were collected into Na-EDTA tubes and then centrifuged to obtain plasma within a maximum of 2 h after withdrawal. The plasma samples were stored at − 86℃ until analysis.

### Analysis of miRNA Expression

#### RNA isolation and cDNA synthesis

Total RNA was isolated from plasma using the miRNeasy Serum/Plasma Kit (Qiagen, Germany) following the manufacturer’s protocol. Spike-in control UniSp6 (Qiagen, Germany) was used to monitor RNA recovery and reverse transcription efficiency. RNA quantities were standardized to 100 ng for cDNA synthesis. The miRCURY LNA RT cDNA synthesis kit (Qiagen, Germany) was used according to the manufacturer's instructions for cDNA synthesis from RNA samples by reverse transcription reaction. The reaction mixture was transferred to a polymerase chain reaction (PCR) device (Eppendorf, Germany). Then, cDNA was stored at −20 ℃ for later use in qRT‒PCR analysis.

#### Quantitative Real-Time PCR (qRT–PCR)

Circulating miRNA expression was analyzed following the SYBR Green-based real-time PCR method on an Applied Biosystems StepOnePlus RT‒PCR instrument (Applied Biosystems, US). We used hsa-miR-24-3p, hsa-miR-181 d-3p, hsa-miR-16-5p (endogenous control), and UniSp6 (spike-in) Qiagen miRCURY PCR primers. All the samples were run in duplicate. Relative expression level (fold change; FC) was calculated following the 2^−ΔΔCt^ method [28]. After the completion of the thermal cycling program, a melting curve analysis was performed to assess the specificity of the amplified products. For quality control, the Ct values for the spike-in control should not exhibit significant deviations across the individual samples.

### Determination of Plasma IFN-γ levels

Plasma IFN-γ was determined using the sandwich ELISA method. A human IFN-γ high-sensitivity ELISA kit (Thermo Fisher, US; catalog number BMS228) with a suitable measurement range of 1.6–100 pg/mL and an analytical sensitivity of 0.99 pg/mL was used. The reaction was measured at 450 nm with an ELISA reader (Thermo Scientific, US). IFN-γ levels (pg/mL) were determined using a standard curve generated by the SkanIT program (version 4.1). The program was also used for blank subtraction before quantifying the IFN-γ concentrations.

### Determination of *IFNG* rs2069727 T/C Genotypes

Genomic DNA was isolated from whole blood samples using a salting-out method. The quantity and purity of the DNA samples were assessed using a NanoDrop One spectrophotometer (Thermo Scientific,US). Genotypes were determined using a TaqMan SNP genotyping assay (Applied Biosystems assay ID rs2069727: C___2683475_10). The reactions were carried out on a StepOnePlus Real-time PCR system (Applied Biosystems, US), and genotype assignments were performed using the allelic discrimination mode of the software (StepOne Software v2.3, Applied Biosystems).

### Statistical Analysis

The distribution of continuous variables (age, plasma IFN-γ, hsa-miR-24-3p, and hsa-miR-181 d-3p expression levels) was assessed using the Kolmogorov–Smirnov test. For the parameters that did not exhibit a normal distribution, the Mann–Whitney *U* test was used for pairwise comparisons, between the following groups: treatment-naïve vs. HCs, GA vs. HCs, and GA vs. treatment-naïve. The Kruskal–Wallis* H* test, on the other hand, was employed for comparisons involving more than two groups. When the Kruskal–Wallis *H* test indicated a significant difference, the Mann–Whitney *U* test with Bonferroni correction was used for post-hoc analyses. Categorical variables, such as gender, genotype, and allele frequency, were expressed as percentages or ratios and compared using the χ2 test. Before the genotype and allele comparisons, the Hardy–Weinberg equilibrium of the SNP was assessed in both the patient and HC groups using the χ2 test. Additionally, balanced odds ratios (ORs), confidence intervals (CIs), and *P* values, adjusted for age and sex, were calculated using the SNPStats program. Receiver operating characteristic (ROC) analyses were used to test the diagnostic value of miRNA expression and ELISA results, and the area under the curve (AUC) was calculated. When statistically significant AUC values were obtained, cutoff points were determined using the Youden index. Then, the sensitivity, specificity, and accuracy were calculated. Since the parameters were not normally distributed, Spearman correlation was used in correlation analysis. Logistic regression analysis was used to determine the association of alleles, genotypes, or phenotypes with MS, and calibration was checked using the Hosmer–Lemeshow test. In pairwise comparisons, the significance limit was accepted as 0.05. In genotype analyses performed with multiple tests, this limit was determined as 0.05/n (n = number of tests), and in other analyses where multiple tests were performed, *P* values ​​were recalculated by the SPSS program as α × n (α = *P* value; n = number of tests performed) with Bonferroni correction. All statistical analyses were performed using IBM SPSS Statistics 26.

## Results

### Demographic and Clinical Characteristics of Patients with RRMS and HCs

Demographic information of the genetic polymorphism group is presented in Table 1. The mean ages of the patients and HCs subjects did not significantly differ. The demographic and clinical information of the patients in the miRNA and ELISA group is also shown in this table. The average age of RRMS patients treated with GA was greater than the average age of HCs (*P* = 0.01), but there was no significant difference in age between the GA treatment and naïve groups or between the treatment-naïve and HC groups. The disease duration (*P* < 0.001) and mean MSSS value (*P* = 0.001) of RRMS patients in the GA group were greater than those of the treatment-naïve group, whereas the number of contrast-enhancing lesions was much lower (*P* = 0.002). When comparing MRI parameters between patient groups, it was observed that the number of T1 lesions (*P* = 0.004) and T2 lesions (*P* = 0.004) were significantly greater in RRMS patients receiving GA treatment compared to treatment-naïve patients. When comparing the frequency of patients who have contrast-enhancing lesions between treatment-naïve and GA-treated RRMS groups, the rate of patients having contrast-enhancing lesions in treatment-naïve RRMS group (%68.0) was significantly greater than that in RRMS patients receiving GA treatment (%24.0) (*P* = 0.002). Most of the GA group (92%) and the entire treatment-naïve group (100%) had mild EDSS (EDSS ≤ 3) scores. When the frequency of RRMS patients with mild (≤ 3) or high and moderate (> 3) EDSS scores was examined, no significant difference was observed between the GA and treatment-naïve groups (*P* = 0.15). Most of the GA and treatment-naïve groups (92% and 96%, respectively) included patients with slow progression (MSSS ≤ 5). The frequency of RRMS patients with slow (MSSS ≤ 5) or rapid (MSSS > 5) progression was examined in terms of treatment, and there was no significant difference (*P* = 0.55) (Table 1).

**Table 1:** Demographic characteristics of the RRMS patient and healthy control groups in the genotyping study and clinical and demographic characteristics of the GA, treatment-naïve, and healthy control groups in the miRNA and ELISA groups

Genetic polymorphism group	**RRMS (*n* = 330)**		**Control (*n* = 330)**	*P*
Age (years), Mean ± SD	34.1 ± 9.3		33.4 ± 10.2	.15^a^
Median (Q1-Q3)	34.0 (26.0–41.0)		31.0 (25.0–39.3)	
Range (min.-max)	42.0 (18.0–60.0)		47.0 (18.0–65.0)	
Sex				.17^b^
Male/Female	114/216		131/199	
Male (%)	34.6		39.7	
miRNA and ELISA group	**GA (*n* = 25)**	**Treatment-naïve (*n* = 25)**	**Control (*n* = 25)**	
Age (years), Mean ± SD	36.4 ± 11.8	31.4 ± 9.5	26.3 ± 4.7	.01^c^
Median (Q1-Q3)	38.0 (25.0–46.0)	31.0 (22.5–38.5)	24.0 (23.0–29.5)	.35^d^
Range (min.-max)	40.0 (21.0–61.0)	31.0 (19.0–50.0)	15.0 (22.0–37.0)	.42^e^ .01^f^
Sex				.14^c^
Male/Female	4/21	10/15	9/16	
Male (%)	16.0	40.0	36.0	
Age at onset (years), Mean ± SD	30.4 ± 10.1	30.8 ± 9.3	-	.90
Median (Q1–Q3)	32.0 (21.5–39.0)	31.0 (19.0–50.0)		
Duration of disease (years), Mean ± SD	5.1 ± 4.5	1.7 ± 4.0	-	<.001
Median (Q1–Q3)	4.0 (1.0–7.0)	0.0 (0.0–1)		
EDSS at onset, Mean ± SD	1.1 ± 1.0	0.7 ± 0.7	-	.17
Median (Q1–Q3)	1.0 (0.5–1.5)	1.0 (0.0- 1.0)		
Mild, EDSS ≤ 3 (n, %)	23 (0.92)	25 (1.0)	-	.15
High and moderate, EDSS > 3 (n, %)	2 (0.08)	0 (0.0)	-	
MSSS, Mean ± SD	1.9 ± 1.9	0.8 ± 1.5	-	.001
Median (Q1–Q3)	1.0 (0.5–1.5)	0.0 (0.0–1.1)		
Slow progression, MSSS ≤ 5 (n, %)	23 (0.92)	24 (0.96)	-	.55
Fast progression, MSSS > 5 (n, %)	2 (0.08)	1 (0.04)	-	
Treatment duration (years), Mean ± SD Median (Q1-Q3)	5.2 ± 5.4 3.0 (1.0–7.0)	-	-	
Number of T1 lesions, Mean ± SD	26.0 ± 14.8	14.0 ± 8.5	-	.004
Median (Q1-Q3)	26.0 (11.3–35.5)	12.0 (8.0–16.5)		
Number of T2 lesions, Mean ± SD	23.0 ± 12.6	13.0 ± 8.6	-	.004
Median (Q1-Q3)	22.5 (11.3–31.5)	11.0 (8.0–14.5)		
Number of contrast-enhancing lesions, Mean ± SD	0.4 ± 1.0	1.4 ± 1.5	-	.002
Median (Q1-Q3)	0.0 (0.0–0.5)	1.0 (0.0–2.0)		
Contrast enhancing lesion (n,%)	6 (24.0)	17 (68.0)	-	.002

^a^Data were compared using the Mann–Whitney U test, RRMS vs. healthy controls; ^b^Data were compared using the χ2 test, RRMS vs. controls. miRNA and ELISA data were expressed as the mean ± SD in the first row and median (Q1–Q3 quartiles) in the second row and were compared using the Kruskal‒Wallis *H* test, and Bonferroni correction was used. ^c^Comparison of the three groups,, ^d^Treatment-naïve RRMS vs. healthy control, ^e^GA vs. naïve, and ^f^GA vs. healthy control

The clinical information of 330 patients with RRMS divided into males and females is summarized in Table 2. There were no significant differences between male and female patients with RRMS in terms of age, age at diagnosis, duration of disease or treatment, or total cholesterol or 25-hydroxyvitamin D_3_ levels. The EDSS (*P* = 0.01) and MSSS (*P* = 0.02) values of male patients with RRMS were greater than those of female patients with RRMS. Most of the study population (93.6%) had mild EDSS (EDSS ≤ 3) scores. When the frequency of RRMS patients with mild (≤ 3), high and moderate (> 3) EDSS scores was examined in terms of sex, a significant difference was observed between male and female patients. The rate of having a high and moderate EDSS score (> 3) was greater in male patients (10.6%) than in female patients (4.2%) (*P* = 0.02). Most of the study population (87.2%) included patients with slowly progressive disease (MSSS value ≤ 5). The numbers of male and female RRMS patients were examined separately in terms of slow or rapid progression of the disease, and a limit line significant sex difference was observed (*P* = 0.05). Triglycerides (*P* < 0.001), low-density lipoprotein cholesterol (LDL-cholesterol) (*P* = 0.04), and very low-density lipoprotein cholesterol (VLDL-cholesterol) (*P* < 0.001) levels were higher in male RRMS patients compared to female RRMS patients. However, high-density lipoprotein cholesterol (HDL-cholesterol) (*P* < 0.001) was significantly lower in male patients than in female patients. Additionally, smoking was significantly more common in male patients (58.8%) than in female patients (16.3%). The DMTs used for male and female patients are summarized in Table 2. The most used treatments for male patients with RRMS were interferon-beta (24.5%) and teriflunomide (24.5%), while the most frequently used treatment for female patients was teriflunomide (24.5%) (Table 2).

**Table 2:** Clinical and demographic information of patients with RRMS categorized by gender

	Male (*n* = 114, %34.55)	Female (*n* = 216, %65.45)	Total (*n* = 330)	*P*
Age (years), Mean ± SD Median (Q1-Q3) Range (min.-max)	34.8 ± 9.6 35.5 (27.0–41.3) 36.0 (18.0–54.0)	33.7 ± 9.2 33.0 (26.0–41.0) 42.0 (18.0–60.0)	34.1 ± 9.3 34.0 (26.0–41.0) 42.0 (18.0–60.0)	.32
Age at onset (years), Mean ± SD Median (Q1–Q3)	29.9 ± 8.8 30.0 (22.0–37.0)	29.5 ± 8.7 27.0 (22.0–36.0)	29.6 ± 8.7 28.0 (22.0–37.0)	.67
Duration of disease (years), Mean ± SD Median (Q1–Q3)	5.0 ± 5.4 3.0 (1.0–8.0)	4.5 ± 4.8 3.0 (1.0- 6.8)	4.7 ± 5.0 3.0 (1.0- 7.0)	.67
EDSS at onset, Mean ± SD Median (Q1–Q3)	1.6 ± 1.2 1.5 (1.0–2.3)	1.2 ± 1.0 1.0 (0.5–2.0)	1.4 ± 1.1 1.0 (0.5–2.0)	.01
Mild, EDSS ≤ 3 (n, %)	101 (89.4)	206 (95.8)	307 (93.6)	.02
High and moderate, EDSS > 3 (n, %)	12 (10.6)	9 (4.2)	21(6.4)	
MSSS, Mean ± SD Median (Q1-Q3)	2.9 ± 2.0 2.4 (1.2–4.3)	2.4 ± 1.8 2.1 (0.9–3.3)	2.6 ± 1.9 2.4 (1.0–3.5)	.02
Slow progression, MSSS ≤ 5 (n, %)	93 (82.3)	193 (89.8)	286 (87.2)	.05
Fast progression, MSSS > 5 (n, %)	20 (17.7)	22 (10.2)	42 (12.8)	
Duration of DMT (years), Mean ± SD	3.9 ± 4.5	3.3 ± 3.8	3.5 ± 4.1	.45
Median (Q1–Q3)	2.0 (0.0–6.0)	2.0 (0.0–5.0)	2.0 (0.0–5.3)	
DMT (n, %)	94 (82.5)	184 (85.2)	278 (84.2)	.84
Interferon-β	23 (24.5)	34 (18.5)	57 (20.5)	.24
Glatiramer acetate	11 (11.7)	33 (17.9)	44 (15.8)	.18
Dimethyl fumarate	12 (12.8)	28 (15.2)	40 (14.4)	.58
Teriflunomide	23 (24.5)	45 (24.5)	68 (24.5)	>.999
Fingolimod	21 (22.3)	36 (19.6)	57 (20.5)	.59
Cladribine	2 (2.1)	2 (1.1)	4 (1.4)	.48
Alemtuzumab	0 (0.0)	1 (0.5)	1 (0.4)	.85
Ocrelizumab	2 (2.1)	4 (2.2)	6 (2.2)	>.999
Rituximab	0 (0.0)	1 (0.5)	1 (0.4)	.36
TG (mmol/L),				
Mean ± SD Median (Q1-Q3)	3.9 ± 2.4 3.0 (2.2–5.1)	3.0 ± 2.3 2.5 (1.8–3.6)	3.3 ± 2.3 2.7 (1.9–4.0)	<.001
Total cholesterol (mmol/L),				
Mean ± SD Median (Q1-Q3)	4.6 ± 1.1 4.6 (3.8–5.4)	4.6 ± 1.0 4.5 (3.9–5.1)	4.6 ± 1.0 4.5 (3.9–5.1)	.84
HDL-cholesterol (mmol/L),				
Mean ± SD Median (Q1-Q3)	1.1 ± 0.2 1.1 (1.0–1.2)	1.4 ± 0.4 1.3 (1.1–1.6)	1.3 ± 0.4 1.2 (1.0–1.5)	<.001
LDL-cholesterol (mmol/L),				
Mean ± SD Median (Q1-Q3))	2.8 ± 1.0 2.7 (2.2–3.2)	2.6 ± 0.8 2.5 (2.0–3.1)	2.7 ± 0.9 2.6 (2.1–3.1)	.04
VLDL-cholesterol (mmol/L),				
Mean ± SD Median (Q1-Q3))	0.8 ± 0.5 0.7 (0.5–1.0)	0.6 ± 0.4 0.5 (0.4–0.7)	0.7 ± 0.5 0.6 (0.4–0.8)	<.001
25-Hydroxyvitamin D (nmol/L),				
Mean ± SD Median (Q1-Q3))	29.3 ± 22.1 21.0 (15.0–34.0)	29.8 ± 22.8 23.0 (15.0–37.0)	29.6 ± 22.6 23.0 (15.0–36.0)	.68
Smoking (Yes, *n* %)	67 (58.8)	35 (16.3)	102 (31.0)	<.001

Data expressed as the mean ± SD in the first row and median (Q1–Q3 quartiles) in the second row were compared using the Mann–Whitney *U* test. Data expressed as frequencies and percentages were compared using the χ2 test. EDSS, Expanded Disability Status Scale; MSSS, Multiple Sclerosis Severity Score; DMT, Disease-Modifying Therapy; TG: triglyceride; HDL, high-density lipoprotein; LDL, low-density lipoprotein; VLDL, very low-density lipoprotein

### *IFNG* rs2069727 T/C Polymorphism Genotype and Allele Frequencies in RRMS Patients and HCs

The genotype and allele frequencies of the *IFNG* rs2069727 T/C polymorphism in the HC and RRMS groups were consistent with HWE (*P* = 0.91 and 0.98, respectively). The *IFNG* rs2069727 T/C SNP was analyzed using SNPstats, adjusting OR, CI, and *P* values for age and sex (Table 3). The frequency of the polymorphic CC genotype was 21.21% in the RRMS group and 24.24% in the HC group. The C allele frequency was 0.456 in RRMS patients and 0.500 in HCs, with no significant difference (*P* = 0.14). In addition, no significant differences were found between groups across different genotype models (dominant: *P* = 0.11; recessive: *P* = 0.44; codominant: *P* = 0.27; overdominant: *P* = 0.44; log-additive: *P* = 0.14). The dominant model was deemed most suitable based on the lowest AIC (917.5) and BIC (935.5) values. Clinical parameters (age at diagnosis, disease duration, EDSS score, MSSS value, and treatment duration) showed no significant differences between RRMS patients when classified by the *IFNG* rs2069727 T/C SNP genotype in both recessive and dominant models (Table 4).

**Table 3:** Genotype and allele frequencies of *IFNG* rs2069727 T/C SNP in RRMS patients and healthy controls

			*Unadjusted*		*Adjusted for age and sex*			
*IFNG* rs2069727 T/C	RRMS (*n* = 200)	Control (*n* = 305)	OR (95% CI)	*P*	OR (95% CI)	*P*	AIC	BIC
Genotype, n (%)			0.84^**a**^ (0.58–1.21) 0.75^**b**^ (0.53–1.05)	.35^**a**^ .10^**b**^	0.86^a^ (0.60–1.25) 0.75^b^ (0.53–1.07) 0.77^d1^ (0.53–1.11) 0.73^d2^ (0.47–1.13) 0.89^e^ (0.65–1.20) 0.85^f^ (0.68–1.06)	.44^c^ .11^b^ .27^d^ .44^e^ .14^f^	919.5^c^ 917.5^b^ 919.4^a^ 919.5^d^ 917.9^e^	937.4^c^ 935.5^b^ 941.9^a^ 937.4^d^ 935.9^e^
TT	99 (30.00)	80 (24.24)						
TC	161 (48.79)	170 (51.52)						
CC	70 (21.21)	80 (24.24)						
Allele frequency								
T	0.544	0.500	0.85^**c**^ (0.68–1.06)	.14^**c**^				
C	0.456	0.500						
HWE *P* Value	.98	.91						

*OR *odds ratio, *CI *confidence interval, *C *cytosine, *T *thymine, *HWE *Hardy–Weinberg Equilibrium, *AIC *Akaike information criterion, *BIC *Bayesian information criterion. Unadjusted *P* & OR values were calculated using the χ^2^ test; Adjusted *P* & OR values were adjusted by sex and age using SNPstat, ^a^Recessive Model. CC vs. TC + TT, ^b^Dominant Model. CC + TC vs. TT, ^c^T vs. C, ^d^Codominant Model, ^d^CC vs. TC vs. TT, ^d1^TC vs. TT, ^d2^CC vs. TT, ^e^Overdominant Model. TC vs. CC + TT, ^f^Log-additive Model. CC vs. TC vs. TT

**Table 4:** Comparison of clinical parameters in RRMS patients classified by *IFNG* rs2069727 T/C SNP genotypes according to recessive and dominant models

	rs2069727 T/C			
Clinical Information	TT (*n* = 99)	TC (*n* = 161)	CC (*n* = 70)	*P*
Age at diagnosis (year), Mean ± SD Median (Q1-Q3)	28.5 ± 8.6 27.0 (22.0–35.0)	30.2 ± 8.6 29.0 (23.5–37.0)	29.8 ± 9.4 27.5(21–37.3)	.95^**a**^ .19^**b**^
Disease duration (year), Mean ± SD Median (Q1-Q3)	5.1 ± 5.2 4.0 (1.0–8.0)	4.8 ± 5.1 3.0 (1.0–7.0)	4.1 ± 4.6 3.0 (0.0–6.0)	.28^**a**^ .38^**b**^
EDSS, Mean ± SD Median (Q1-Q3)	1.3 ± 1.0 1.0 (1.0–2.0)	1.3 ± 1.1 1.0 (0.5–2.0)	1.4 ± 1.1 1.5 (0.5–2.0)	.61^**a**^ .80^**b**^
MSSS, Mean ± SD Median (Q1-Q3)	2.4 ± 1.8 2.0 (1.1–3.5)	2.5 ± 1.8 2.3 (1.0–3.2)	2.9 ± 2.0 2.4 (1.0–4.3)	.16^**a**^ .51^**b**^
Treatment duration (year), Mean ± SD Median (Q1-Q3)	3.8 ± 3.9 3.0 (0.0–6.0)	3.6 ± 4.3 2.0 (0.0–6.0)	3.0 ± 3.8 2.0 (0.0–4.0)	.19^**a**^ .26^**b**^

Data expressed as mean ± standard deviation (SD) in the first row and median (Q1–Q3 quartiles) in the second row were compared using the Mann–Whitney *U* test. For the recessive model

^**a**^CC vs. TC and TT, for the dominant model

^**b**^CC and TC vs. TT. EDSS, Expanded Disability Status Scale; MSSS, Multiple Sclerosis Severity Score

### Plasma miRNA Expression and IFN-γ Concentration in Untreated and GA-Treated RRMS Patients and HCs

**Fig. 1:**
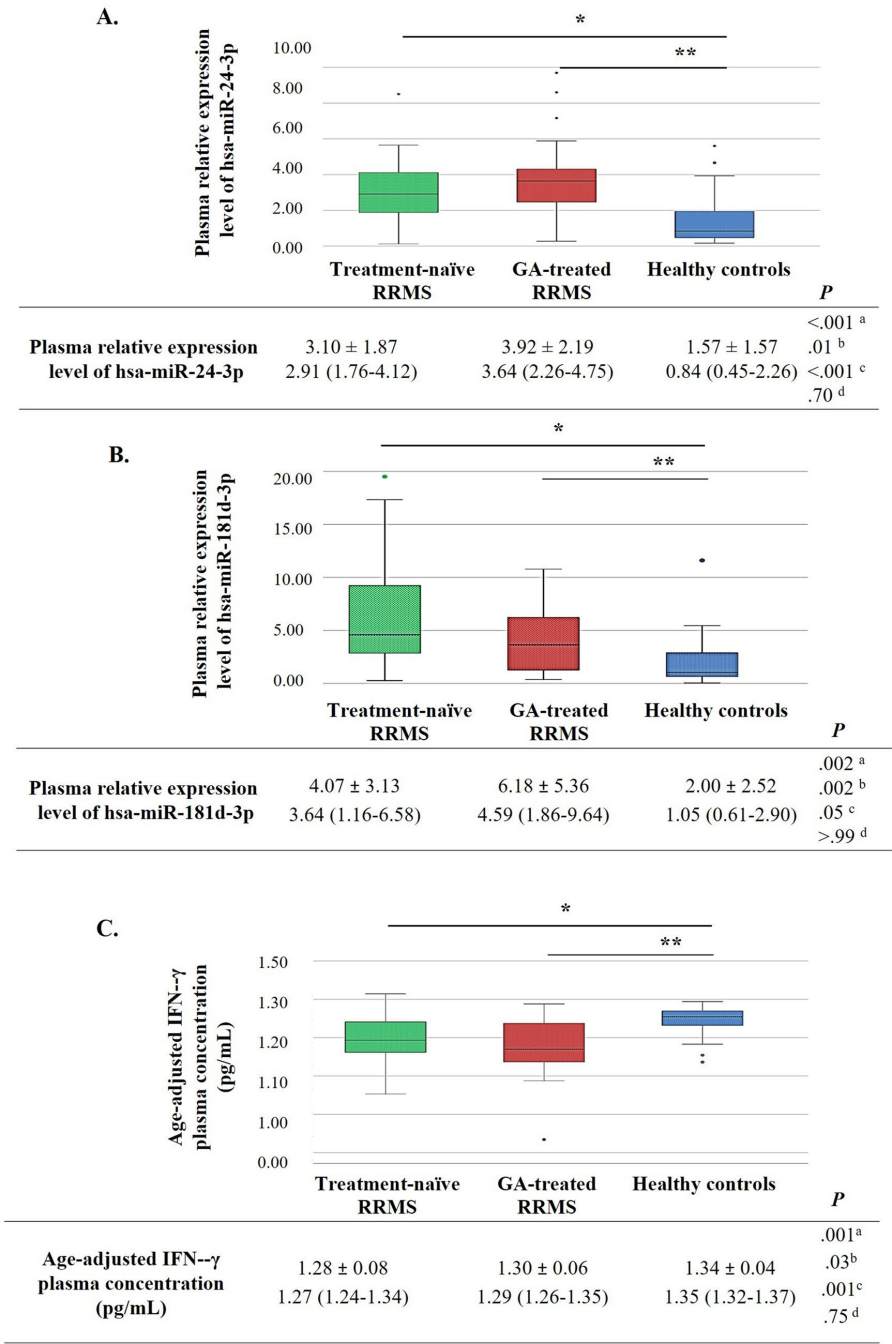
Comparison of plasma hsa-miR-24-3p (**A**) and hsa-miR-181 d-3p relative expression (**B**) and age-adjusted IFN-γ levels (pg/mL) (**C**) among the GA-treated, treatment-naïve, and healthy control groups. IFN-γ: interferon-γ. GA: glatiramer acetate. The prefix hsa (homo sapiens) is used to denote humans. Data in the embedded tables A, B, and C expressed as the mean±SD in the first row and median (Q1–Q3 quartiles) in the second row were compared using the Kruskal‒Wallis *H* test. Post-hoc analyses were performed with the Mann-Whitney *U* test, and Bonferroni correction was implemented. ^a ^3 groups, ^b ^treatment-naïve RRMS vs. control, ^c ^GA vs. control and, ^d ^GA vs. treatment-naïve RRMS. For A, *: *P* =.01, **:*P* <.001; for B, *:*P* =.002, **:*P* =.05; for C, *:*P* =.03, **:*P* <.001

Circulating miRNA expression levels were determined in plasma samples from the miRNA and ELISA group. Analysis of the differences in miRNA expression between the groups revealed that hsa-miR-24-3p was overexpressed in treatment-naïve and GA-treated RRMS groups compared to the HC group (treatment-naïve RRMS vs. HC: *P* = 0.01; GA-treated RRMS vs. HC: *P* < 0.001) (Fig. 1A). In addition, the relative expression of hsa-miR-181 d-3p increased significantly in treatment-naïve and GA-treated RRMS groups compared to the HC group (treatment-naïve RRMS vs. HC: *P* = 0.002; GA-treated RRMS vs. HC: *P* = 0.05) (Fig. 1B).

Besides, a comparison between untreated (naïve) and GA-treated RRMS patients was performed, but significant differences in miRNA expression levels were not detected (Fig. 1A and B).

**Fig. 2:**
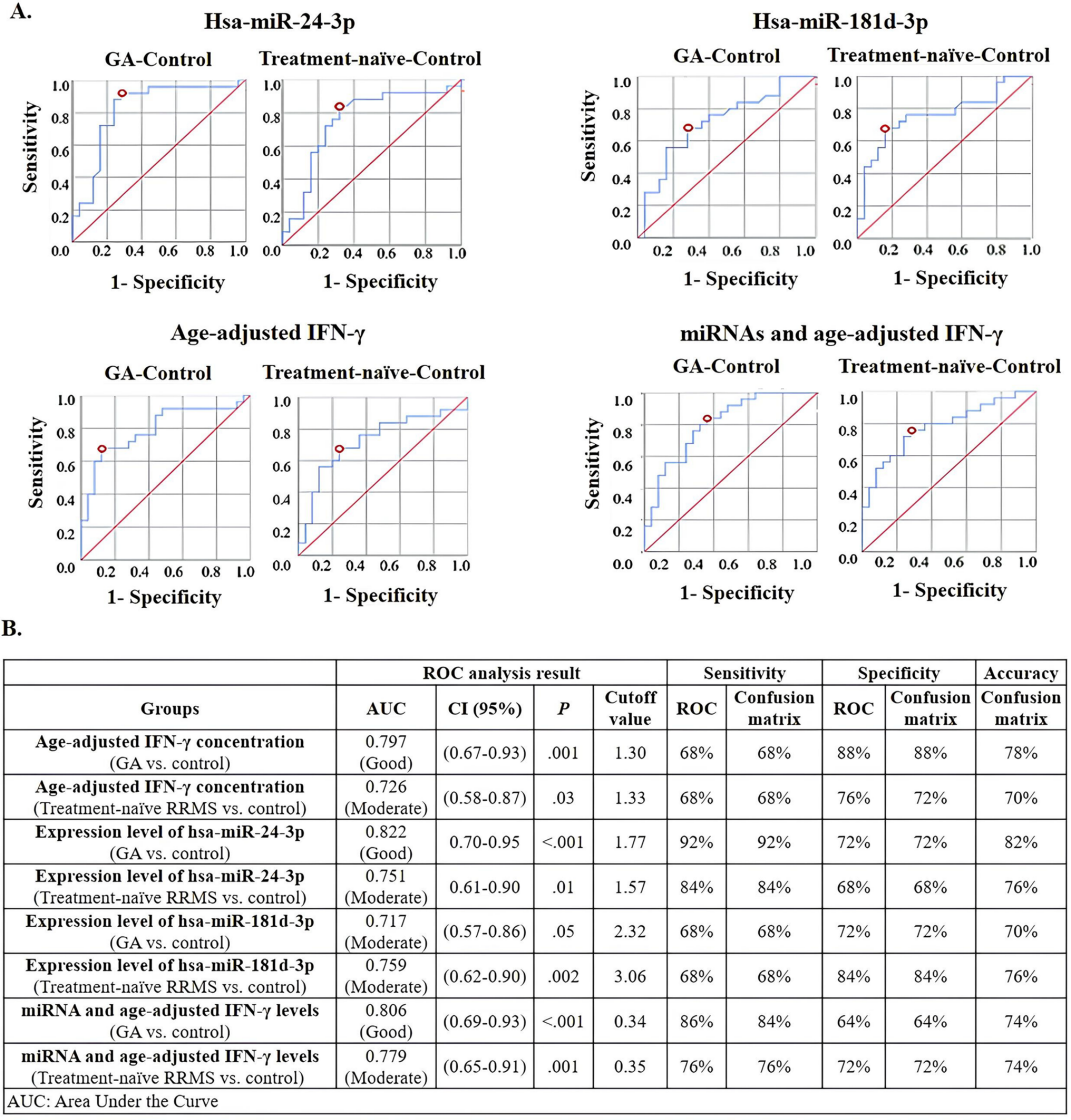
ROC curves (**A**) and ROC curves’ analysis and error matrix results (**B**) of plasma hsa-miR-24-3p and hsa-miR-181 d-3p relative expression levels and age-adjusted plasma IFN-γ concentrations (pg/mL). Blue line: plasma levels of the parameters; red line: reference line; red circle: cutoff value. IFN-γ: interferon-γ. GA: glatiramer acetate

Linear regression analysis was used to adjust plasma IFN-γ levels for age. We observed that the age-adjusted plasma IFN-γ levels decreased significantly in treatment-naïve and GA-treated RRMS groups compared to the HC group (treatment-naïve vs. HC: *P* = 0.03, GA vs. HC: *P* = 0.001) (Fig. 1C). Since there were differences between groups in terms of relative expression levels and age-adjusted IFN-γ levels, ROC analysis was performed to determine the diagnostic performance of these parameters and to determine their potential as clinical diagnostic markers (Fig. 2A and B).

The diagnostic performance of plasma hsa-miR-24-3p relative expression level was categorized as moderate (AUC = 0.751, *P* = 0.01, 95% CI = 0.61–0.90, sensitivity = 84%, specificity = 68%, accuracy = 76%, cutoff = 1.57) in distinguishing treatment-naïve RRMS patients from HC individuals. The diagnostic performance of this parameter in distinguishing RRMS patients receiving GA treatment from HC individuals was classified as good (AUC = 0.822, *P* < 0.001, 95% CI = 0.70–0.95, sensitivity = 92%, specificity = 72%, accuracy = 82%, cutoff = 1.77) based on ROC analysis. Similarly, the diagnostic performance of hsa-miR-181 d-3p for categorizing treatment-naïve RRMS patients from HC individuals was classified as moderate (AUC = 0.759, *P* = 0.002, 95% CI = 0.62–0.90, sensitivity = 68%, specificity = 84%, accuracy = 76%, cutoff = 3.06. The diagnostic performance of this parameter in distinguishing RRMS patients receiving GA treatment from HC individuals was deemed moderate (AUC = 0.717, *P* = 0.05, 95% CI = 0.57–0.86, sensitivity = 68%, specificity = 72%, accuracy = 70%, cutoff = 2.32) in ROC analysis (Fig. 2B).

Furthermore, the diagnostic performance of plasma age-adjusted IFN-γ level in distinguishing RRMS patients from HC individuals was classified as moderate (AUC = 0.726, *P* = 0.03, 95% CI = 0.58–0.87, sensitivity = 68%, specificity = 76%, accuracy = 70%, cutoff = 1.33). Moreover, the diagnostic performance of plasma age-adjusted IFN-γ level in distinguishing RRMS patients receiving GA treatment from HC individuals was considered good (AUC = 0.797, *P* = 0.001, 95% CI = 0.67–0.93, sensitivity = 68%, specificity = 88%, accuracy = 78%, cutoff = 1.30) based on ROC analysis (Fig. 2B).

Additionally, we evaluated plasma age-adjusted IFN-γ level, the relative expression levels of hsa-miR-24-3p and hsa-miR-181 d-3p collectively as a single parameter, and found out that the diagnostic performance of this single parameter, in distinguishing RRMS patients from HC individuals was categorized as moderate (AUC = 0.779, *P* = 0.001, 95% CI = 0.65–0.91, sensitivity = 76%, specificity = 72%, accuracy = 74%, cutoff = 0.35). The diagnostic performance of this single parameter, in distinguishing RRMS patients receiving GA treatment from HC individuals was classified as good (AUC = 0.806, *P* = 0.001, 95% CI = 0.69–0.93, sensitivity = 86%, specificity = 64%, accuracy = 74%, cutoff = 0.34) based on ROC analysis (Fig. 2B).

### Examination of Plasma IFN-γ Levels According to *IFNG* rs2069727 T/C Genotypes

Statistical analysis of age-adjusted mean plasma IFN-γ levels by genotype showed no significant differences among wild homozygote (TT) and polymorphic homozygote (CC) individuals across the GA-treated RRMS, treatment-naïve RRMS, and HC groups (*P* = 0.48 and *P* = 0.21, respectively), while heterozygote (TC) individuals had significantly lower IFN-γ levels in the GA-treated RRMS group compared to HCs (*P* = 0.01).

**Fig. 3:**
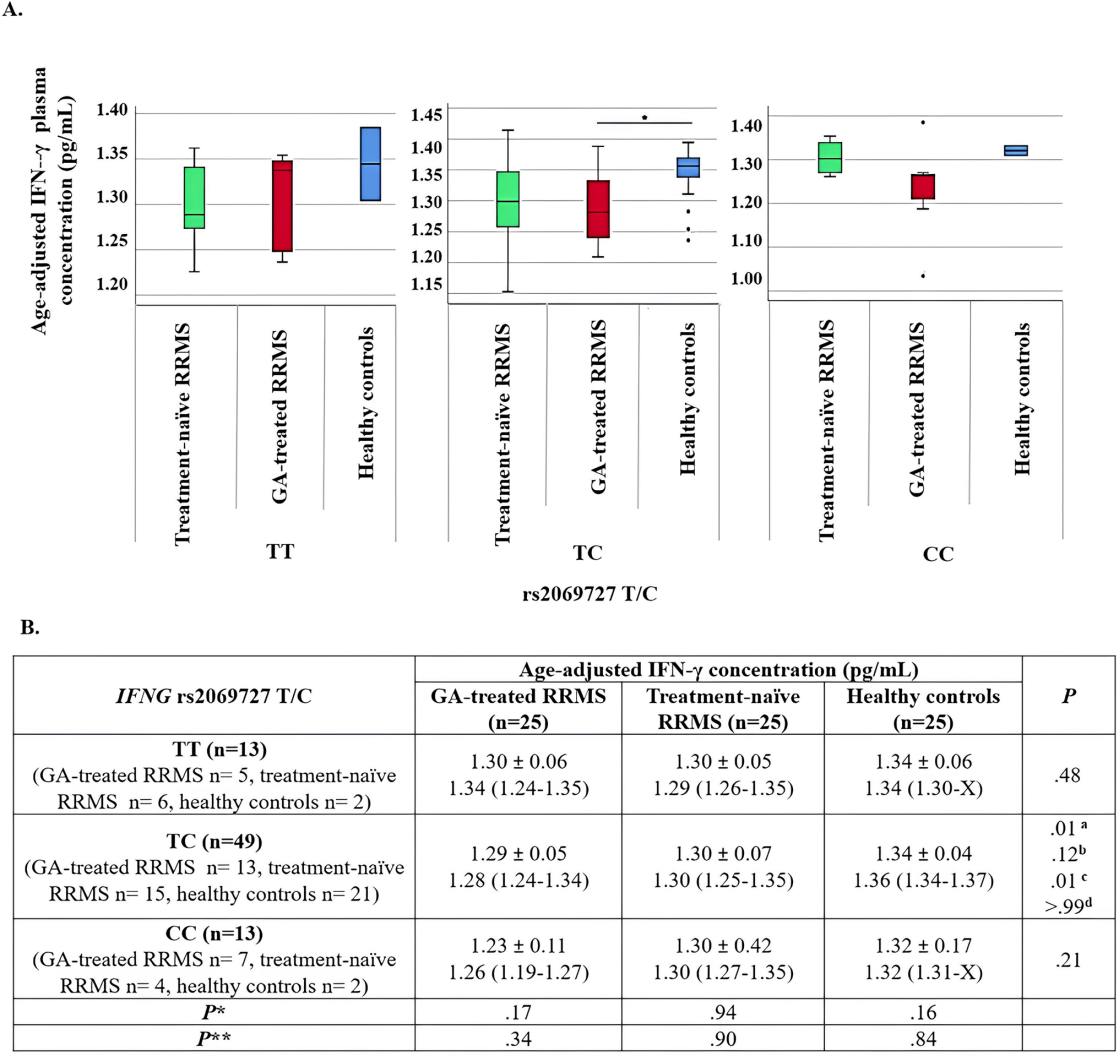
Plasma age-adjusted IFN-γ concentrations in *IFNG* rs2069727 T/C genotype groups:(A) box plots illustrating the distribution of IFN-γ levels, including the median (central line), interquartile range (box), and potential outliers (individual points beyond the whiskers). (B) table summarizing the age-adjusted IFN-γ concentrations (pg/mL) as mean ± standard deviation (SD) and median (Q1-Q3). Since the number of people was < 3 in some groups, 'X' was written instead of the Q3 value. The Kruskal-Wallis-*H* test was used for comparison between the three groups, and when significant results were obtained, post-hoc analyses were performed with the Mann-Whitney *U* test, and Bonferroni correction was used. ^a^ 3 groups, ^b^ Treatment-naïve RRMS vs. healthy controls, ^c^ GA-treated RRMS vs. healthy controls, and ^d^ GA-treated RRMS vs. treatment-naïve RRMS. *Recessive model: CC vs. TC and TT, and **Dominant model: CC and TC vs. TT. Pairwise comparison analysis was performed using the Mann-Whitney *U* test. For A, *:P=.01. GA: glatiramer acetate. IFN-γ: Interferon-γ

To assess the impact of *IFNG* rs2069727 T/C genotypes on age-adjusted plasma IFN-γ levels, comparisons within each study group (GA-treated RRMS, treatment-naïve RRMS, and HCs) were conducted using recessive and dominant genetic models. No significant differences were observed in the GA-treated RRMS group (recessive model: *P* = 0.17; dominant model: *P* = 0.34); treatment-naïve RRMS group (recessive model: *P* = 0.94; dominant model: *P* = 0.90) and HCs group (recessive model: *P* = 0.16; dominant model: *P* = 0.84) between polymorphic homozygote (CC) and wild homozygote (TT) or heterozygote (TC) individuals (Fig. 3A and B).

### Examination of the Relationships Between hsa-miR-24-3p, hsa-miR-181 d-3p, and IFN-γ According to Breakpoints

The median values of plasma hsa-miR-24-3p and hsa-miR-181 d-3p relative expression levels and age-adjusted plasma IFN-γ levels were determined as limit values (FC = 2.61, 2.90, and 1.31 pg/mL, respectively). The relationship between them based on the limit values has been investigated, and significant differences have been observed. According to the analysis of the whole group, individuals in the low IFN-γ (≤ 1.31 pg/mL) group exhibited greater relative hsa-miR-24-3p expression (mean ± SD = 3.30 ± 1.91) than did individuals in the high IFN-γ (> 1.31 pg/mL) group (mean ± SD = 2.42 ± 2.24) (*P* = 0.02). Moreover, the mean plasma IFN-γ level in individuals in the low hsa-miR-181 d-3p (≤ 2.90) group (mean ± SD = 1.32 ± 0.05 pg/mL) was greater than that in individuals in the high hsa-miR-181 d-3p (> 2.90) group (mean ± SD = 1.29 ± 0.07 pg/mL) (*P* = 0.02). The relative expression levels of plasma hsa-miR-24-3p and hsa-miR-181 d-3p in individuals with high IFN-γ concentrations (> 1.31 pg/mL) were compared between groups, and significantly greater levels of hsa-miR-24-3p and hsa-miR-181 d-3p were detected in the GA group than in the HC group (GA vs. HC: *P* = 0.01 for both). The age-adjusted plasma IFN-γ levels of individuals with high plasma hsa-miR-181 d-3p relative expression levels (> 2.90) were greater than those of patients in the GA group (mean ± SD = 1.25 ± 0.08 pg/mL), patients in the treatment-naïve group (mean ± SD = 1.31 ± 0.06 pg/mL), and individuals in the HC group (mean ± SD = 1.33 ± 0.03 pg/mL) (*P* = 0.02). In the group of individuals with high plasma hsa-miR-181 d-3p relative expression levels, lower age-adjusted IFN-γ levels were detected in patients in the GA group than in HCs (*P* = 0.04) (Table 5).

**Table 5:** The relationship between age-adjusted plasma IFN-γ level, plasma hsa-miR-24-3p and hsa-miR-181 d-3p relative expression levels according to limit values

	GA-treated RRMS (*n* = 25)	Treatment-naïve (TN) RRMS (*n* = 25)	Control (*n* = 25)	Total (*n* = 75)	*P*
IFN-γ ≤ 1.311 (GA n = 17, TN n = 15, Control = 6)	**hsa-miR-24-3p**				
	3.90 ± 2.05 3.93 (2.47–4.75)	3.25 ± 1.51 3.07 (2.23–4.12)	1.71 ± 1.69 1.19 (0.36–3.13)	3.30 ± 1.91 3.31 (2.05–4.25)	.07
IFN-γ > 1.311 (GA n = 19, TN n = 10, Control = 8)	3.97 ± 2.63 3.18 (2.11–5.29)	2.88 ± 2.38 2.32 (1.19–4.11)	1.53 ± 1.58 0.84 (0.47–19.4)	2.42 ± 2.24 1.88 (0.66–3.43)	.009^**a**^ .23^**b**^ .01^**c**^ .77^**d**^
P*	.59	.37	.93	.02	
IFN-γ ≤ 1.311 (GA n = 17, TN n = 15, Control = 6)	**hsa-miR-181 d-3p**				
	4.49 ± 2.97 3.81 (2.26–6.58)	4.53 ± 3.58 3.15 (0.81–8.09)	2.22 ± 1.99 2.06 (0.51–3.65)	4.15 ± 3.15 3.62 (1.16–6.29)	.27
IFN-γ > 1.311 (GA n = 19, TN n = 10, Control = 8)	3.16 ± 3.46 1.81 (0.71–6.35)	8.66 ± 6.74 8.09 (2.58–14.62)	1.94 ± 2.72 0.92 (0.55–2.27)	4.02 ± 5.08 1.57 (0.70–6.04)	.01^**a**^ .23^**b**^ .01^**c**^ .77^**d**^
P*	.24	.10	.56	.20	
hsa-miR-24-3p ≤ 2.61 (GA n = 7, TN n = 11, Control = 20)	**IFN-γ**				
	1.26 ± 0.12 1.26 (1.21–1.35)	1.29 ±.07 1.30 (1.24–1.35)	1.34 ±.04 1.35 (1.33–1.37)	1.31 ±.08 1.34 (1.26–1.36)	.05
hsa-miR-24-3p > 2.61 (GA n = 18, TN n = 14, Control = 5)	1.28 ± 0.05 1.27 (1.24–1.33)	1.31 ±.05 1.30 (1.26–1.34)	1.34 ±.04 1.35 (1.30–1.38)	1.30 ±.05 1.28 (1.26–1.34)	.05
P*	.84	.65	.92	.12	
hsa-miR-181 d-3p ≤ 2.90 (GA n = 11, TN n = 8, Control = 19)	**IFN-γ**				
	1.31 ±.06 1.33 (1.24–1.35)	1.29 ±.05 1.27 (1.26–1.34)	1.35 ±.04 1.36 (1.34–1.37)	1.32 ±.05 1.34 (1.26–1.37)	.04^**a**^ .06^**b**^ .19^**c**^ >.99^**d**^
hsa-miR-181 d-3p > 2.90 (GA n = 14, TN n = 17, Control = 6)	1.25 ±.08 1.26 (1.23–1.28)	1.31 ±.06 1.31 (1.27–1.35)	1.33 ±.03 1.32 (1.30–1.36)	1.29 ±.07 1.28 (1.25–1.34)	.02^**a**^ .07^**b**^ .04^**c**^ >.99^**d**^
P*	.12	.34	.12	.02	

The parameters are presented as mean ± standard deviation (SD) in the first row and median (Q1-Q3) in the second row. The'n'denotes the number of individuals corresponding to the given parameter. The comparison among the three groups was conducted using the Kruskal–Wallis *H* test. Post-hoc analyses were performed using the Mann–Whitney U test with Bonferroni correction when a significant result was obtained. The P* values obtained from pairwise comparisons were determined using the Mann–Whitney *U* test, ^**a**^3 groups, ^**b**^treatment-naïve RRMS vs. healthy control, ^**c**^GA-treated vs. healthy control, and, ^**d**^GA-treated vs. treatment-naïve RRMS. GA = Glatiramer acetate, TN = treatment-naïve. IFN-γ = interferon-γ

### Correlation Analysis

In the GA group, age positively correlated with EDSS score (ρ = 0.511, *P* = 0.009), disease duration (ρ = 0.642, *P* = 0.001), and treatment duration (ρ = 0.643, *P* = 0.001). The EDSS score positively correlated with MSSS value (ρ = 0.788, *P* < 0.001), disease duration (ρ = 0.598, *P* = 0.002), and treatment duration (ρ = 0.699, *P* < 0.001), and negatively correlated with age-adjusted plasma IFN-γ levels (ρ = −0.520, *P* = 0.008). Disease duration correlated positively with treatment duration (ρ = 0.862, *P* < 0.001) and negatively with plasma IFN-γ levels (ρ = −0.596, *P* = 0.002). Plasma IFN-γ levels negatively correlated with treatment duration (ρ = −0.623, *P* = 0.001). In treatment-naïve RRMS patients, MSSS values correlated positively with EDSS scores (ρ = 0.395, *P* = 0.05) and disease duration (ρ = 0.895, *P* < 0.001). In treatment-naïve RRMS patients, relative expression levels of hsa-miR-24-3p and hsa-miR-181 d-3p were positively correlated (ρ = 0.458, *P* = 0.02). In the HC group, hsa-miR-24-3p and hsa-miR-181 d-3p expression levels also showed a positive correlation (ρ = 0.607, *P* = 0.001).

### Logistic Regression Analysis

The models created for logistic regression analyses are presented in Table 6. Logistic regression analysis revealed that the relative expression level of plasma hsa-miR-181 d-3p (Table 7) was a significant predictor of RRMS vs. HC status (OR = 1.520, CI = 1.091–2.116, *P* = 0.01). In the logistic regression results between GA-treated RRMS and treatment-naïve RRMS, GA treatment was associated with the relative expression level of plasma hsa-miR-181 d-3p (OR = 0.789, CI = 0.628–0.992, *P* = 0.04). Furthermore, logistic regression analysis revealed that the composite parameter, age-adjusted plasma IFN-γ levels, relative plasma hsa-miR-24-3p expression level, and relative hsa-miR-181 d-3p expression level when evaluated as a single parameter, was a significant predictor of RRMS status compared with the HC status (OR = 1.127, CI = 1.021–1.245, *P* = 0.02) (Table 7).

**Table 6:** The models of logistic regression analyses

Model A (covering analyses conducted in the genotype group): Independent variables: Age, gender, rs2069727 T/C genotype	Model A.1 Analysis between RRMS and control group		Model A.1.1 Recessive Model
			Model A.1.2 Dominant Model
Model B (covering analyses of miRNA expression levels): Model B.1 (Each parameter was evaluated separately) Independent variables: Plasma hsa-miR-24-3p, hsa-miR-181 d-3p relative expression levels, age-adjusted plasma IFN-γ level, rs2069727 T/C genotype, MSSS, LDL cholesterol, HDL cholesterol, triglycerides, total lesion count, contrast-enhancing lesion count. Model B.1 consists of two different sections: B.1.a including the lipid panel and B.1.b excluding it	Model B.1.a The model includes lipid parameters	Model B.1.a.1 GA vs. control group	Model B.1.a.1.1 Recessive Model
			Model B.1.a.1.2 Dominant Model
		Model B.1.a.2 GA vs. treatment-naïve group	Model B.1.a.2.1 Recessive Model
			Model B.1.a.2.2 Dominant Model
		Model B.1.a.3 Treatment-naïve vs. control group	Model B.1.a.3.1 Recessive Model
			Model B.1.a.3.2 Dominant Model
	Model B.1.b Model without lipid parameters	Model B.1.b.1 GA vs. control group	Model B.1.b.1.1 Recessive Model
			Model B.1.b.1.2 Dominant Model
		Model B.1.b.2 GA vs. treatment-naïve group	Model B.1.b.2.1 Recessive Model
			Model B.1.b.2.2 Dominant Model
		Model B.1.b.3 Treatment-naïve vs. control group	Model B.1.b.3.1 Recessive Model
			Model B.1.b.3.2 Dominant Model
Model B.2 (Plasma hsa-miR-24-3p and hsa-miR-181 d-3p relative expression levels and age-adjusted plasma IFN-γ level were examined as a single parameter) Independent variables: Plasma hsa-miR-24-3p, hsa-miR-181 d-3p relative expression levels, and age-adjusted plasma IFN-γ level, rs2069727 T/C genotype, MSSS, LDL cholesterol, HDL cholesterol, triglycerides, total lesion count, contrast-enhancing lesion count Model B.2 consists of two different sections: B.2.a including the lipid panel and B.2.b excluding it	Model B.2.a The model includes lipid parameters	Model B.2.a.1 GA vs. control group	Model B.2.a.1.1 Recessive Model
			Model B.2.a.1.2 Dominant Model
		Model B.2.a.2 GA vs. treatment-naïve group	Model B.2.a.2.1 Recessive Model
			Model B.2.a.2.2 Dominant Model
		Model B.2.a.3 Treatment-naïve vs. control group	Model B.2.a.3.1 Recessive Model
			Model B.2.a.3.2 Dominant Model
	Model B.2.b Model without lipid parameters	Model B.2.b.1 GA vs. control group	Model B.2.b.1.1 Recessive Model
			Model B.2.b.1.2 Dominant Model
		Model B.2.b.2 GA vs. treatment-naïve group	Model B.2.b.2.1 Recessive Model
			Model B.2.b.2.2 Dominant Model
		Model B.2.b.3 Treatment-naïve vs. control group	Model B.2.b.3.1 Recessive Model
			Model B.2.b.3.2 Dominant Model

HDL, high-density lipoprotein; LDL, low-density lipoprotein; VLDL, very low-density lipoprotein; MSSS, multiple sclerosis severity score; GA, glatiramer acetate. IFN-γ: Interferon-γ

**Table 7:** Statistically significant results from logistic regression analyses of treatment-naïve vs. healthy control and GA vs. treatment-naïve groups

Treatment-naïve vs. Healthy Controls	Associated parameters	*P*	OR	95% CI	Hosmer– Lemeshow goodness of fit test		
					χ2	df	*P*
Model B.1.a.3, Model B.1.a.3.1, Model B.1.a.3.2	Plasma hsa-miR-181 d-3p relative expression levels are associated with RRMS	.01	1.520	1.091–2.116	4.612	8	.80
Model B.1.b.3, Model B.1.b.3.1, Model B.1.b.3.2		.005	1.348	1.096–1.659	5.861	8	.66
Model B.2.a.3, Model B.2.a.3.1 Model B.2.a.3.2	Plasma hsa-miR-24-3p and hsa-miR-181 d-3p relative expression levels, evaluated as a single parameter with age-adjusted IFN-γ levels, are associated with RRMS	.02	1.127	1.021–1.245	7.727	8	.46
Model B.2.b.3, Model B.2.b.3.1, Model B.2.b.3.2	Plasma hsa-miR-24-3p and hsa-miR-181 d-3p relative expression levels, evaluated as a single parameter with IFN-γ levels, are associated with RRMS	.008	1.072	1.018–1.129	6.446	8	.60
Model B.1.a.3, Model B.1.a.3.1, Model B.1.a.3.2	Plasma hsa-miR-181 d-3p relative expression levels are associated with RRMS	.01	1.520	1.091–2.116	4.612	8	.80
Model B.1.b.3, Model B.1.b.3.1, Model B.1.b.3.2		.005	1.348	1.096–1.659	5.861	8	.66
Model B.2.a.3, Model B.2.a.3.1 Model B.2.a.3.2	Plasma hsa-miR-24-3p and hsa-miR-181 d-3p relative expression levels, evaluated as a single parameter with age-adjusted IFN-γ levels, are associated with RRMS	.02	1.127	1.021–1.245	7.727	8	.46
Model B.2.b.3, Model B.2.b.3.1, Model B.2.b.2.2	Plasma hsa-miR-24-3p and hsa-miR-181 d-3p relative expression levels, evaluated as a single parameter with IFN-γ levels, are associated with RRMS	.008	1.072	1.018–1.129	6.446	8	.60
GA vs. Treatment-naïve	**Associated parameters**	***P***	**OR**	**95% CI**	**Hosmer– Lemeshow goodness of fit test**		
					**χ2**	**df**	***P***
Model B.1.a.2, Model B.1.a.2.1, Model B.1.a.2.2	GA treatment is associated with plasma hsa-miR-181 d-3p relative expression levels	.04	0.789	0.628–0.992	1.28	8	>.99

The analyses were conducted using binary logistic regression with backward LR selection method, with the two subgroups divided into Model A (RRMS group n = 330, healthy control n = 330) for the genotype group and Model B (Glatiramer acetate (GA) n = 25, treatment-naïve n = 25, and healthy control n = 25) for miRNA expression level group. Due to the lack of significant associations found in logistic regression analyses for Model A, the results for Model A are not presented in the table. OR: Odds ratio, CI: Confidence interval

## Discussion

In this study, the IFN-γ levels in plasma, the *IFNG* rs2069727 T/C variation, which is thought to affect this level, and the relative expression levels of hsa-miR-24-3p and hsa-miR-181 d-3p were examined in terms of their contribution to MS risk and disability scores. The interrelationships of these parameters were also examined to determine whether they had a strong relationship with the disease, and their utility as biomarkers was evaluated.

The *IFNG* rs2069727 T/C SNP was previously studied in MS and was found to be associated with the IFN-γ expression level and MS risk in the American (US population-based), Northern Irish, and Belgian cohorts. It was determined that men carrying the C allele for this SNP had a greater expression level than those who did not carry this allele (*P* = 0.002) [16]. It is known that allele frequencies vary between populations and that MS shows polygenic inheritance. In our population, the frequency of the polymorphic C allele did not differ significantly between the RRMS (0.456) and HC (0.500) groups. No significant difference was observed in the statistical evaluation of genotype distribution between groups in any genotype model.

It was determined that the relative expression levels of plasma hsa-miR-24-3p and hsa-miR-181 d-3p were significantly higher in both treatment-naïve and GA groups compared to HCs. No significant difference was found between the GA and treatment-naïve groups. Several studies have also shown increased hsa-miR-24-3p expression levels in patients with MS compared to HCs [29–32]. Ehya et al. [29] found significantly higher hsa-miR-24-3p levels in MS patients (RRMS, primary progressive MS (PPMS), and SPMS patients were examined together) compared to HCs (*P* < 0.01) (2017). Vistbakka et al. [31] reported increased hsa-miR-24-3p in RRMS and PPMS patients versus HCs (*P* = 0.01) and a positive correlation with EDSS scores (r = 0.264, *P* = 0.03). Our study confirmed higher hsa-miR-24-3p levels in RRMS patients compared to HCs. ROC analysis for diagnostic value of hsa-miR-24-3p in treatment-naïve RRMS patients showed an AUC of 0.751, indicating moderate diagnostic performance (*P* = 0.01). However, when these analyses are interpreted in conjunction with the results obtained from previous studies, it is evident that hsa-miR-24-3p levels are significantly higher not only in the RRMS subtype [31], but also in the group including patients with PPMS [30, 32] and in the mixed group containing PPMS, SPMS, and RRMS patients compared to HCs (*P* < 0.01) [25].

Currently, the role of miR-181 d-3p in MS is poorly understood. Fayyad-Kazan et al. reported that miR-24-3p and miR-181 d-3p negatively regulate the expression of IFN-γ mRNA and protein by binding to target regions in the 3'UTR of *IFNG* mRNA [20]. In our study, ROC curve analysis was performed to evaluate the diagnostic power of relative plasma hsa-miR-181 d-3p expression. In comparing treatment-naïve RRMS patients and HCs, the AUC was 0.759, indicating moderate diagnostic performance (*P* = 0.002). These findings suggest that elevated plasma hsa-miR-181 d-3p levels can serve as a potential biomarker with moderate diagnostic utility (sensitivity = 68%, specificity = 84%, accuracy = 76%) to distinguish RRMS patients from HCs.

Many studies in the literature indicate that the expressions of hsa-miR-24-3p and hsa-miR-181 d-3p change with diseases such as cancer, cardiovascular disease, Alzheimer's disease and Parkinson's disease, and these levels may vary even in different stages of the same disease [31–33]. The role and expression levels of these miRNAs in specific diseases are not yet fully understood, requiring further research to evaluate their relationships with diseases.

The target region of miR-24-3p and miR-181 d-3p is in the 3'UTR of *IFNG* mRNA [20]. In a prior study, miR-24-3p was shown to negatively regulate IFN-γ mRNA and protein expression by binding to this target region [20]. In our study, it turns out that the age-adjusted plasma IFN-γ level of patients in treatment-naïve and GA-treated RRMS groups was significantly lower than that of the HCs (treatment-naïve vs. HC: *P* = 0.03; GA vs. HC: *P* = 0.001). There was no significant difference in the age-adjusted plasma IFN-γ concentration between the GA group and the treatment-naïve group. The age-adjusted plasma IFN-γ level could be a test with moderate diagnostic value (AUC = 0.726, *P* = 0.03) when comparing treatment-naïve patients with HCs, with a sensitivity of 68%, a specificity of 72%, and an accuracy of 70%. According to these analyses, the decreased age-adjusted plasma IFN-γ levels found in patient plasma samples can be considered a potential biomarker with moderate diagnostic value, as it is a feature that distinguishes the RRMS subtype from HCs.

Within the scope of this study, the association between age-adjusted plasma IFN-γ concentration and the *IFNG* rs2069727 T/C SNP was examined using recessive and dominant models, and no association with genotypes was observed in treatment-naïve RRMS patients, RRMS patients receiving GA treatment or HCs. On the contrary, in the literature, a study conducted by Kantarci et al. [16] examined the association between genotypes of this SNP and IFN-γ levels in peripheral blood mononuclear cells (PBMCs). In their study, it was found that males carrying the rs2069727*G allele exhibited higher *IFNG* gene expression compared to individuals with other genotypes. The relationship between genotypes of the rs2069727 T/C SNP and age-adjusted plasma IFN-γ concentration has been investigated for the first time in MS patients in Turkey.

**Fig. 4:**
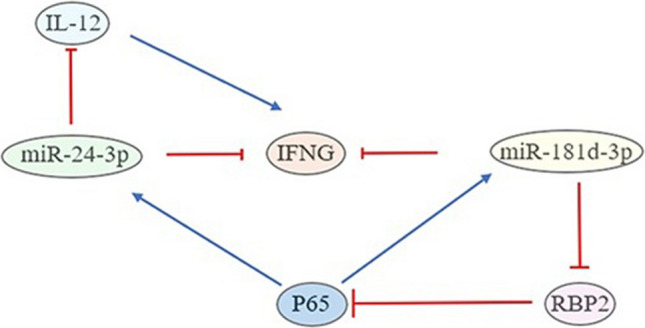
Molecular factors that affect/are affected by hsa-miR-24-3p, hsa-miR-181 d-3p, and IFN-γ levels (in this network created according to data in the literature, the blue indicator represents upregulation; the red indicator represents suppression of expression). Nuclear factor of kappa light polypeptide gene enhancer in B-Cells 3 (*P65*), retinol-binding protein 2 (*RBP2*), interleukin 12 (*IL12*), interferon-γ (*IFNG*)

Existing evidence indicates that the transcription factor nuclear factor of kappa light polypeptide gene enhancer in B-Cells 3 (p65) exhibits increased expression in immune cells infiltrating the CNS as well as PBMCs of RRMS patients [34]. Importantly, p65 overexpression has been shown to significantly upregulate miR-181 d-3p expression, while p65 inhibition greatly downregulates miR-181 d-3p by suppressing its promoter activity. Additionally, miR-181 d-3p can inhibit p65 through a feedback mechanism [35]. Moreover, p65 has been reported to upregulate the expression of miR-23a, miR-27a, and miR-24-3p [36]. Collectively, these studies suggest that the elevated p65 levels in RRMS patients may indirectly contribute to decreased IFN-γ concentrations by driving high miR-24-3p and miR-181 d-3p expression. This is further supported by the finding that miR-181 d-3p can directly inhibit the p65 suppressor retinol-binding protein 2 (*RBP2*), leading to an increased p65 expression [35]. Notably, the cytokine interleukin 12 (IL-12), which upregulates IFN-γ, is negatively regulated by miR-24-3p in an in vitro study using cluster of differentiation-14 monocytes [37]. Taken together with the observed elevation of hsa-miR-24-3p in RRMS patients compared to HCs, this suggests that high hsa-miR-24-3p levels may directly inhibit *IFNG* mRNA and downregulate IL-12, ultimately leading to reduced IFN-γ concentrations (Fig. 4).

In our study, there was a statistically significant difference in hsa-miR-24-3p and hsa-miR-181 d-3p levels between RRMS patients receiving GA treatment and HCs, with greater levels observed in the GA treatment group. Since any parameter whose expression changes with the disease is expected to return to normal levels found in HC individuals because of treatment [37], our study's surprising GA vs. HCs comparison results may be attributed to the cross-sectional nature of the study rather than a longitudinal design. Moreover, plasma levels of hsa-miR-24-3p and hsa-miR-181 d-3p were significantly elevated in treatment-naïve RRMS patients compared to HCs (*P* = 0.01 and *P* = 0.002, respectively). With GA treatment, these levels were expected to decrease compared to treatment-naïve patients; hsa-miR-24-3p expression did not decrease (*P* = 0.70) and reduction was not statistically significant for hsa-miR-181 d-3p (*P* > 0.99). Age-adjusted plasma IFN-γ levels were significantly lower in GA-treated RRMS patients compared to HCs (*P* = 0.001) and also lower in treatment-naïve patients compared to HCs (*P* = 0.03). The anticipated increase in IFN-γ levels with GA treatment, noted for its neuroprotective effects, was not observed. Possible explanations for this outcome include inter-individual variability inherent to the cross-sectional design and the possibility that GA's immunomodulatory effects primarily target other pathways or cytokines not examined in this study.

Unlike Ehya et al.'s findings [29], our study found no significant correlation between EDSS and hsa-miR-24-3p expression. In the GA group, no significant correlation was found between the relative expression of hsa-miR-24-3p or hsa-miR-181 d-3p and any other parameters. However, in treatment-naïve and HCs groups, a positive correlation existed between hsa-miR-24-3p and hsa-miR-181 d-3p. This correlation was absent in GA-treated RRMS patients, suggesting that the pharmacodynamic effects of GA may alter miRNA interactions.

Age-adjusted plasma IFN-γ levels were negatively correlated with the EDSS score, disease duration, and treatment duration in both the GA-treated RRMS and the combined group of the GA-treated and treatment-naïve RRMS patients. In addition, plasma IFN-γ levels were decreased in the treatment-naïve and GA groups compared to HCs. This finding suggests that a reduction in IFN-γ plasma levels may correlate with greater disability, highlighting a potential association between plasma IFN-γ levels and disease progression in patients undergoing GA therapy. This suggests inadequate immune suppression in patient groups, potentially contributing to the observed disease progression and worsening disability scores. Notably, the GA-treated RRMS patients exhibited persistently low IFN-γ levels, despite this cytokine being known to promote immune suppression in later disease stages [10, 24]. This was accompanied by an increase in MSSS values (*P* = 0.001) and no improvement in EDSS scores. These findings suggest potential associations between IFN-γ levels and treatment outcomes, highlighting the need for further investigation through longitudinal studies. The possible reasons for these results are as follows:

GA treatment has been shown to exert both neuroprotective and immunomodulatory effects. In GA-treated patients, unlike in HCs, it has been demonstrated that activated GA-reactive Th2 cells produce not only protective Th2 cytokines such as IL-4, IL-5, and IL-13 but also protective neurotrophic factors such as insulin-like growth factor-1 (IGF-1), IGF-2, and brain-derived neurotrophic factor (BDNF). The distinct cytokine profiles of GA-induced T cells versus HCs may contribute to differential protein, mRNA, and miRNA expression patterns in RRMS patients receiving GA treatment [38]. Additionally, GA can competitively inhibit the activation of myelin-specific T cells, leading to distinct effects compared to HCs. The elevated levels of hsa-miR-24-3p and hsa-miR-181 d-3p observed in GA-treated RRMS patients versus HCs may be attributed to sequential changes in the composition of innate, adaptive, and GA-specific T cell populations. Notably, miR-24-3p regulates T cell proliferation and Th1/Th17 differentiation, while miR-181 d-3p modulates T cell maturation and cytokine expression [20, 39

In this study, the relative expression levels of the miRNAs were examined and the age-adjusted plasma IFN-γ concentration were evaluated together as a single parameter. To distinguish treatment-naïve RRMS patients from HC, the combined parameter demonstrated better diagnostic power (AUC = 0.779, moderate) compared to when the parameters were considered individually. Moreover, to distinguish GA-treated RRMS patients from HCs, the single combined parameter demonstrated better discriminatory power (AUC = 0.806, good) compared to the individual parameters of hsa-miR-181 d-3p relative expression level (AUC = 0.717, moderate) and age-adjusted IFN-γ concentration (AUC = 0.797, good). However, it showed lower discriminatory power than the hsa-miR-24-3p expression level parameter (AUC = 0.822, good). These findings suggest that this combined parameter could be a potential research subject for larger studies investigating the pharmacodynamic effects of GA.

In our study, the relationships between age-adjusted plasma IFN-γ levels and plasma hsa-miR-24-3p and hsa-miR-181 d-3p relative expression levels were investigated based on median limit values. When interpreted alongside the interactions shown in Fig. 4, the findings suggest that hsa-miR-24-3p and hsa-miR-181 d-3p negatively influence IFN-γ protein levels, either directly or indirectly.

The limitations of the study include the lack of longitudinal data to observe changes over time and the relatively small sample size in the subgroup. Nonetheless, the sample size was statistically adequate.

## Conclusion

In this study, the *IFNG* rs2069727 T/C SNP was examined for the first time in Turkey concerning the risk of MS. Additionally, there is no other study in the literature on the plasma relative expression levels of hsa-miR-181 d-3p in MS. Furthermore, this study is the first to evaluate IFN-γ protein levels and the levels of hsa-miR-24-3p and hsa-miR-181 d-3p, which are thought to affect this level, together in terms of RRMS diagnosis and GA treatment response.

Consequently, there is still a need to expand the gene repertoire whose expression changes with GA treatment. Besides, further longitudinal studies will shed light on the overlooked aspects of GA's pharmacodynamic effect.

In our study, it was observed that the (age-adjusted) plasma IFN-γ concentration and the relative expression levels of hsa-miR-24-3p and hsa-miR-181 d-3p have the potential to serve as blood-based biomarkers for RRMS. Additionally, plasma IFN-γ concentrations were also analyzed in patients undergoing GA therapy. The lack of increase in IFN-γ in this group, along with the increase in MSSS values, and the lack of improvement in EDSS scores, warrants further investigation into possible associations between IFN-γ and treatment outcomes. There was a negative correlation between IFN-γ levels and EDSS scores in GA therapy group, which suggests that a decrease in IFN-γ plasma levels may correlate with greater disability, potentially indicating a link between IFN-γ plasma levels and disease progression in patients receiving GA therapy. However, due to the cross-sectional nature of this study, the interpretation of this potential is limited. Longitudinal studies would provide a broader perspective on the relationship between plasma IFN-γ levels and GA treatment, shedding light on its impact on disease progression and treatment response. Moreover, the biomarker candidates revealed by this research will contribute to the development of more effective diagnostic methods, future biosensor technologies, and new treatment options.

## Data Availability

Data is provided within the manuscript or supplementary information files.

## References

[1] Kister I, Chamot E, Salter AR, Cutter GR, Bacon TE, Herbert J (2013) Disability in multiple sclerosis: A reference for patients and clinicians. Neurology 80(11):1018–1024. 10.1212/WNL.0b013e3182872855 23427319 10.1212/WNL.0b013e3182872855 PMC3653203

[2] Briggs FBS, Acuna B, Shen L, Ramsay P, Quach H, Bernstein A, Bellesis KH, Kockum IS et al (2014) Smoking and risk of multiple sclerosis: Evidence of modification by NAT1 variants. Epidemiology 25(4):605–614. 10.1097/EDE.0000000000000089 24625537 10.1097/EDE.0000000000000089

[3] Graves MC, Benton M, Lea RA, Boyle M, Tajouri L, Macartney-Coxson D, Scott RJ, Lechner-Scott J (2014) Methylation differences at the HLA-DRB1 locus in CD4+ T-Cells are associated with multiple sclerosis. Mult Scler J 20(8):1033–1041. 10.1177/1352458513516529 10.1177/1352458513516529 24336351

[4] Regev K, Healy BC, Paul A, Diaz-Cruz C, Mazzola MA, Raheja R, Glanz BI, Kivisäkk P et al (2018) Identification of MS-specific serum miRNAs in an international multicenter study. Neurol: Neuroimmunol NeuroInflamm 5(5):e491. 10.1212/NXI.0000000000000491 30175165 10.1212/NXI.0000000000000491 PMC6117192

[5] Bingen JM, Clark LV, Band MR, Munzir I, Carrithers MD (2023) Differential DNA methylation associated with multiple sclerosis and disease modifying treatments in an underrepresented minority population. Front Genet 13:1058817. 10.3389/fgene.2022.1058817 36685876 10.3389/fgene.2022.1058817 PMC9845287

[6] Shirvani-Farsani Z, Kakhki MP, Gargari BN, Doosti R, Moghadasi AN, Azimi AR, Behmanesh M (2017) The expression of VDR mRNA but not NF-κB surprisingly decreased after vitamin D treatment in multiple sclerosis patients. Neurosci Lett 653:258–263. 10.1016/j.neulet.2017.05.050 28576565 10.1016/j.neulet.2017.05.050

[7] Kantarci OH (2019) Phases and Phenotypes of Multiple Sclerosis. CONTINUUM: Lifelong Learn Neurol 25(3):636–654. 10.1212/con.0000000000000737 10.1212/CON.0000000000000737 31162309

[8] Efendi H, Yandım Kuşcu D. Multiple Skleroz Tanı ve Tedavi Kılavuzu (2018) Available from: www.galenos.com.tr. Accessed 21 Jun, 2023

[9] Finkelsztejn A (2014) Multiple sclerosis: Overview of Disease-Modifying agents. Perspect Med Chem 6:65–72. 10.4137/PMC.S13213 10.4137/PMC.S13213 PMC4197902 25336899

[10] Wagner CA, Roqué PJ, Goverman JM (2020) Pathogenic T cell cytokines in multiple sclerosis. J Exp Med 217(1):1–10. 10.1084/jem_20190460 31611252 10.1084/jem.20190460 PMC7037255

[11] Young HA, Hodge DL (2003) Interferon-γ. In: Encyclopedia of Hormones, Elsevier, , pp 391–397. 10.1016/B0-12-341103-3/00151-0

[12] Simpson S, Stewart N, Van Der Mei I, Otahal P, Charlesworth J, Ponsonby AL, Blizzard L, Dwyer T et al (2015) Stimulated PBMC-produced IFN-γ and TNF-α are associated with altered relapse risk in multiple sclerosis: Results from a prospective cohort study. J Neurol Neurosurg Psychiatry 86(2):200–207. 10.1136/jnnp-2013-307336 24790215 10.1136/jnnp-2013-307336

[13] Shokrgozar MA, Sarial S, Amirzargar A, Shokri F, Rezaei N, Arjang Z, Radfar J, Yousefi-Behzadi M et al (2009) IL-2, IFN-γ, and IL-12 gene polymorphisms and susceptibility to multiple sclerosis. J Clin Immunol 29(6):747–751. 10.1007/s10875-009-9310-z 19543959 10.1007/s10875-009-9310-z

[14] Arellano G, Ottum PA, Reyes LI, Burgos PI, Naves R (2015) Stage-specific role of interferon-gamma in experimental autoimmune encephalomyelitis and multiple sclerosis. Front Immunol 6(SEP):29. 10.3389/fimmu.2015.00492 26483787 10.3389/fimmu.2015.00492 PMC4586507

[15] Montalvo Villalba MC, Valdés Ramírez O, Muné Jiménez M, Arencibia Garcia A, Martinez Alfonso J, González Baéz G, Roque Arrieta R, Rosell Simón D et al (2020) Interferon gamma, TGF-β1 and RANTES expression in upper airway samples from SARS-CoV-2 infected patients. Clin Immunol 220:108576. 10.1016/j.clim.2020.108576 32866645 10.1016/j.clim.2020.108576 PMC7455570

[16] Kantarci OH, Hebrink DD, Schaefer-Klein J, Sun Y, Achenbach S, Atkinson EJ, Heggarty S, Cotleur AC et al (2008) Interferon gamma allelic variants: Sex-biased multiple sclerosis susceptibility and gene expression. Arch Neurol 65(3):349–357. 10.1001/archneurol.2007.66 18332247 10.1001/archneurol.2007.66

[17] Weber JA, Baxter DH, Zhang S, Huang DY, Huang KH, Lee MJ, Galas DJ, Wang K (2010) The MicroRNA Spectrum in 12 Body Fluids. Mol Diagn Genet. 10.1373/clinchem.2010.147405 10.1373/clinchem.2010.147405 PMC4846276 20847327

[18] Mansoor S, Ghasemi-Kasman M, Yavarpour-Bali H (2020) The role of microRNAs in multiple sclerosis. Int Rev Immunol. 10.1080/08830185.2020.1826474 32997552 10.1080/08830185.2020.1826474

[19] miRWalk (2024) miRWalk database, from http://mirwalk.umm.uni-heidelberg.de/. Accessed: 20 January, 2024

[20] Fayyad-Kazan H, Hamade E, Rouas R, Najar M, Fayyad-Kazan M, El Zein N, ElDirani R, Hussein N et al (2014) Downregulation of microRNA-24 and -181 parallels the upregulation of IFN-γ secreted by activated human CD4 lymphocytes. Hum Immunol 75(7):677–685. 10.1016/j.humimm.2014.01.007 24704866 10.1016/j.humimm.2014.01.007

[21] Fordham JB, Afsar RN, Nares S (2015) miR-24 Regulates Macrophage Polarization and Plasticity. J Clin Cell Immunol 176(3):139–148. 10.1053/j.gastro.2016.08.014.CagY 10.4172/2155-9899.1000362 PMC4721581 26807309

[22] Chen YY, Ho HL, Lin SC, Ho TDH, Hsu CY (2018) Upregulation of miR-125b, miR-181d, and miR-221 Predicts Poor Prognosis in MGMT Promoter-Unmethylated Glioblastoma Patients. Am J Clin Pathol 149(5):412–417. 10.1093/ajcp/aqy008 29538610 10.1093/ajcp/aqy008

[23] Hohol MJ, Orav EJ, Weiner HL (1999) Disease steps in multiple sclerosis: A longitudinal study comparing Disease Steps and EDSS to evaluate disease progression. Mult Scler 5(5):349–354. 10.1177/135245859900500508 10516779 10.1177/135245859900500508

[24] Melnikov M, Sharanova S, Sviridova A, Rogovskii V, Murugina N, Nikolaeva A, Dagil Y, Murugin V et al (2020) The influence of glatiramer acetate on Th17-immune response in multiple sclerosis. PLoS One 15(10 October):e0240305. 10.1371/journal.pone.0240305 33126239 10.1371/journal.pone.0240305 PMC7599084

[25] Thompson AJ, Banwell BL, Barkhof F, Carroll WM, Coetzee T, Comi G, Correale J, Fazekas F et al (2018) Diagnosis of multiple sclerosis: 2017 revisions of the McDonald criteria. Lancet Neurol 17(2):162–173. 10.1016/S1474-4422(17)30470-2 10.1016/S1474-4422(17)30470-2 29275977

[26] Kurtzke J (1983) Rating neurologic impairment in multiple sclerosis: an expanded disability status scale (EDSS). Neurology 33(11):1444–52 6685237 10.1212/wnl.33.11.1444

[27] Roxburgh RHSR, Seaman SR, Masterman T, Hensiek AE, Sawcer SJ, Vukusic S, Achiti I, Confavreux C et al (2005) Multiple sclerosis severity score: Using disability and disease duration to rate disease severity. Neurology 64(7):1144–1151. 10.1212/01.WNL.0000156155.19270.F8 15824338 10.1212/01.WNL.0000156155.19270.F8

[28] Livak KJ, Schmittgen TD (2001) Analysis of relative gene expression data using real-time quantitative PCR and the 2-ΔΔCT method. Methods 25(4):402–408. 10.1006/meth.2001.1262 11846609 10.1006/meth.2001.1262

[29] Ehya F, Tehrani HA, Garshasbi M, Nabavi SM (2017) Identification of miR-24 and miR-137 as novel candidate multiple sclerosis miRNA biomarkers using multi-staged data analysis protocol. Mol Biol Res Commun 6(3):127–140. 10.22099/mbrc.2017.24861.1256 29071282 10.22099/mbrc.2017.24861.1256 PMC5640895

[30] Vistbacka J (2022) Circulating microRNAs as biomarkers for multiple sclerosis. J Neurol Sci. https://trepo.tuni.fi/handle/10024/141742. Accessed 01.02.2024

[31] Vistbakka J, Elovaara I, Lehtimäki T, Hagman S (2017) Circulating microRNAs as biomarkers in progressive multiple sclerosis. Mult Scler 23(3):403–412. 10.1177/1352458516651141 27246141 10.1177/1352458516651141

[32] Vistbakka J, Sumelahti ML, Lehtimäki T, Elovaara I, Hagman S (2018) Evaluation of serum miR-191-5p, miR-24-3p, miR-128-3p, and miR-376c-3 in multiple sclerosis patients. Acta Neurol Scand 138(2):130–136. 10.1111/ane.12921 29527713 10.1111/ane.12921

[33] Burgos K, Malenica I, Metpally R, Courtright A, Rakela B, Beach T, Shill H, Adler C et al (2014) Profiles of extracellular miRNA in cerebrospinal fluid and serum from patients with Alzheimer’s and Parkinson’s diseases correlate with disease status and features of pathology. PLoS One 9(5):e94839. 10.1371/journal.pone.0094839 24797360 10.1371/journal.pone.0094839 PMC4010405

[34] Eggert M, Goertsches R, Seeck U, Dilk S, Neeck G, Zettl UK (2008) Changes in the activation level of NF-kappa B in lymphocytes of MS patients during glucocorticoid pulse therapy. J Neurol Sci 264(1–2):145–150. 10.1016/j.jns.2007.08.026 17889033 10.1016/j.jns.2007.08.026

[35] Zhou M, Yin X, Zheng L, Fu Y, Wang Y, Cui Z, Gao Z, Wang X et al (2021) miR-181d/RBP2/NF-κB p65 Feedback Regulation Promotes Chronic Myeloid Leukemia Blast Crisis. Front Oncol 11(March):1–12. 10.3389/fonc.2021.654411 10.3389/fonc.2021.654411 PMC8027495 33842368

[36] Zu LD, Peng XC, Zeng Z, Wang JL, Meng LL, Shen WW, Hu CT, Yang Y et al (2018) Gastrin inhibits gastric cancer progression through activating the ERK-P65-miR23a/27a/24 axis. J Exp Clin Cancer Res 37(1):1–18. 10.1186/s13046-018-0782-7 29866191 10.1186/s13046-018-0782-7 PMC5987590

[37] Afsar R, Naqvi JBF, Nares S (2015) miR-24 Regulates Macrophage Polarization and Plasticity. J Clin Cell Immunol 06(05):362. 10.4172/2155-9899.1000362 10.4172/2155-9899.1000362 PMC4721581 26807309

[38] Ganji A, Ebrahimi M, Shapoori S, Nourbakhsh P, Ghazavi A (2020) Cytokine effects of interferon and glatiramer acetate on cytokine patterns in multiple sclerosis patients. Cytokine 126(October 2019):154911. 10.1016/j.cyto.2019.154911 31731047 10.1016/j.cyto.2019.154911

[39] Pua HH, Steiner DF, Patel S, Gonzalez JR, Ortiz-Carpena JF, Kageyama R, Chiou NT, Gallman A et al (2016) MicroRNAs 24 and 27 suppress allergic inflammation and target a network of regulators of T helper 2 cell-associated cytokine production. Immunity 44(4):821–832. 10.1016/j.immuni.2016.01.003 26850657 10.1016/j.immuni.2016.01.003 PMC4838571

